# Severe Sepsis After Vaginal Delivery: A Case Report

**DOI:** 10.1155/crog/9666897

**Published:** 2026-06-18

**Authors:** Maryam Dehghan

**Affiliations:** ^1^ Obstetrician and Gynecologist, Alzahra Hospital, Isfahan University of Medical Sciences, Isfahan, Iran, mui.ac.ir

**Keywords:** hysterectomy, maternal morbidity, postpartum sepsis, septic shock, source control

## Abstract

**Background:**

Complications of pregnancy and childbearing are one of the most important public health concerns worldwide. Postpartum sepsis remains a leading cause of maternal morbidity and mortality. Early recognition and timely intervention, including surgical source control, when necessary, are critical for improving outcomes.

**Case Presentation:**

We report a 29‐year‐old gravida 2 woman with a history of opioid use, malnutrition, and very low socioeconomic status. She presented 10 days after a preterm vaginal delivery at 24 weeks′ gestation with severe sepsis. She developed refractory septic shock, with evidence of endometritis, salpingitis, empyema, and renal microabscesses. Blood cultures grew methicillin‐resistant *Staphylococcus aureus* (MRSA). Despite aggressive fluid resuscitation and broad‐spectrum antibiotics, her condition deteriorated, necessitating a total abdominal hysterectomy with bilateral salpingectomy and chest tube insertion on postpartum Day 14. The patient improved postoperatively and was discharged 14 days after surgery.

**Discussion:**

This case highlights the importance of early recognition of postpartum sepsis, rapid initiation of empiric broad‐spectrum antibiotics, and multidisciplinary management. Risk factors such as comorbidities, malnutrition, and low socioeconomic status may increase disease severity and complicate management. In cases unresponsive to medical therapy, surgical source control can be lifesaving, even following vaginal delivery.

**Conclusion:**

Clinicians should maintain a low threshold for aggressive intervention in high‐risk postpartum patients. Early diagnosis, multidisciplinary care, and timely surgical intervention are key to reducing maternal morbidity and mortality.

## 1. Introduction

Complications of pregnancy and childbearing are one of the most important public health concerns globally. Approximately 810 women die every day from preventable causes related to pregnancy in the world [1]. About 60% of maternal deaths occur during delivery and the immediate postpartum period. Meanwhile, sepsis, defined as life‐threatening organ dysfunction caused by a dysregulated host response to infection, is one of the main causes of severe maternal morbidity and maternal death [2]. Maternal mortality increases at the same time as sepsis‐related maternal death, possibly due to several increased risk factors for sepsis such as cesarean delivery—the most common factor—as well as smoking, comorbidity disease, malnutrition, and obesity [3]. Pregnant and peripartum women are at risk of developing sepsis due to modulated immune system during these periods. In addition, the physiological changes of pregnancy can mimic and sometimes mask diagnostic criteria of sepsis, so the recognition of an infection can be delayed until progression to severe sepsis [4]. As a result, an increasing severity of infection occurs, which correlates with an increased mortality rate: 16.7% for sepsis, 25%–30% for severe sepsis, and up to 40%–70% for septic shock in the general population [2].

Recent case reports and small case series have documented the need for emergent hysterectomy as definitive surgical source control in postpartum patients with refractory septic shock, severe endometritis, or uterine necrosis unresponsive to medical management [5, 6]. However, the majority of reported cases involve cesarean delivery or early postpartum deterioration [5, 7]; delayed presentations following vaginal delivery remain rarely described.

Furthermore, contemporary literature highlights that postpartum sepsis requiring hysterectomy is more likely in the presence of comorbid conditions such as substance use disorder, malnutrition, anemia, and limited access to prenatal care, yet these factors remain underrepresented in published case reports [6]. The present case adds to the limited evidence by describing delayed‐onset postpartum septic shock after preterm vaginal delivery, caused by a multidrug‐resistant pathogen in a woman with a history of opioid use, malnutrition, and very low socioeconomic status, in which hysterectomy was a life‐saving intervention, underscoring the importance of early multidisciplinary decision‐making and timely surgical source control.

Given that this topic is clinically relevant and addresses an important cause of maternal morbidity and mortality, we aim to emphasize the importance of identifying underlying risk factors, early recognition, and aggressive management of maternal sepsis.

## 2. Case Presentation

A 29‐year‐old gravida 2 woman presented to our hospital 10 days after a preterm vaginal delivery at 24 weeks′ gestation with progressive systemic symptoms, including fever, weakness, and decreased level of consciousness. She had significant risk factors for infection, including opioid use disorder, severe malnutrition, very low socioeconomic status, and lack of prenatal care.

On admission, she appeared acutely ill and lethargic. Vital signs showed hypotension (BP: 80/50 mmHg), fever (38°C), tachycardia (125 beats/min), tachypnea (30 breaths/min), and oxygen saturation of 92% on room air. Physical examination revealed generalized edema without abdominal tenderness or abnormal vaginal discharge. Laboratory evaluation demonstrated leukocytosis (WBC 22,000/*μ*L; neutrophils 89%), severe anemia (Hb 7.7 g/dL), elevated ESR and CRP, acute kidney injury (Cr 2 mg/dL), and hypoalbuminemia (1.9 g/dL).

Initial imaging included chest radiography, showing bilateral pleural effusions. Echocardiography revealed mild pericardial effusion without valvular abnormalities. Abdominopelvic CT showed hypodensity in the lower uterine segment and mild pelvic fluid without intra‐abdominal abscess.

Empiric broad‐spectrum intravenous antibiotics were initiated immediately: vancomycin 1 g every 12 h and meropenem 1 g every 8 h, along with aggressive fluid resuscitation and vasopressor support. Despite therapy, she remained febrile and hypotensive. On postpartum Day 12, transvaginal ultrasonography revealed endometritis and bilateral salpingitis. Pleural ultrasound demonstrated empyema, and renal ultrasound revealed bilateral microabscesses. Blood cultures grew methicillin‐resistant *Staphylococcus aureus* (MRSA), likely related to injection drug use, a recognized risk factor for postpartum MRSA bacteremia.

Due to persistent septic shock unresponsive to medical therapy, a multidisciplinary team recommended surgical source control. On postpartum Day 14, she underwent total abdominal hysterectomy with bilateral salpingectomy and chest tube insertion. Antibiotics were continued postoperatively, tailored to culture sensitivities (vancomycin continued for a total of 21 days, meropenem de‐escalated after clinical improvement). The patient improved gradually and was discharged on postpartum Day 28, 14 days after surgery.

## 3. Discussion

Maternal sepsis is defined as a life‐threatening organ dysfunction resulting from a dysregulated host response to infection, whereas septic shock represents a severe subset with profound circulatory, cellular, and metabolic abnormalities that markedly increase mortality risk [5]. Physiologic adaptations of pregnancy, including a hyperdynamic circulatory state, low systemic vascular resistance, hypoalbuminemia, and increased intravascular fluid requirements, may exacerbate tissue edema and organ dysfunction during sepsis, as observed in our patient [3].

Epidemiological studies demonstrate that progression from uncomplicated sepsis to severe sepsis or septic shock is influenced by both medical and social risk factors. Analysis of over 1.6 million live births in California revealed that advanced maternal age, primiparity, multiple gestation, minority race/ethnicity, low education, public or absent insurance, and comorbid conditions such as diabetes and chronic hypertension were associated with increased risk of severe maternal sepsis [4]. Larger population studies similarly identify preterm birth, anemia, cesarean section, retained products of conception, and postpartum hemorrhage as significant risk factors. Additional determinants such as home delivery, repeated vaginal examinations, and rural residence have been highlighted in low‐resource settings [6, 7]. In addition, injection drug use that is a proven risk factor for *S. aureus* infections in the general population appears as a common risk factor for this type of bacteremia in pregnant patients with high rates of poor outcomes in recent case reports such as our patient [8, 9]. Our patient presented with multiple vulnerabilities, including anemia, preterm delivery, malnutrition, low socioeconomic status, opioid use, and absence of prenatal care, likely contributing to the severity of her septic presentation.

While empiric broad‐spectrum antimicrobial therapy and fluid resuscitation are the cornerstones of maternal sepsis management, timely source control is critical when infection persists or progresses despite medical therapy [3]. Case reports and small series from the last 5 years describe postpartum hysterectomy as a life‐saving intervention in refractory septic shock due to endometritis, necrotizing uterine infection, or multidrug‐resistant pathogens, typically following cesarean delivery or early postpartum deterioration [4, 5, 8]. Our case differs in that septic shock developed after vaginal delivery, with MRSA bacteremia likely linked to injection drug use, illustrating a rare but clinically significant scenario.

Decision‐making regarding surgical intervention must balance the patient′s hemodynamic stability, the likelihood of achieving source control, and the risks of operative intervention. Case reports in the literature further underscore the role of surgical intervention in severe postpartum infections. For example, Lemierre‐like syndrome involving endomyometritis leading to hysterectomy illustrates how aggressive surgical source control may be lifesaving when severe infection and thrombosis are present [10]. Necrotizing soft tissue infections after childbirth have also been described where hysterectomy or extensive debridement was necessary due to progression despite antibiotics [11, 12] These reports, along with observational data, support an emerging role for early surgical decision‐making in refractory cases. In this case, persistent hemodynamic instability, evidence of disseminated infection (endometritis, salpingitis, empyema, and renal microabscesses), and failure to respond to broad‐spectrum antibiotics prompted a multidisciplinary decision for total abdominal hysterectomy with bilateral salpingectomy. The favorable outcome reinforces that early recognition of refractory infection and timely surgical source control are essential to reduce maternal morbidity and mortality.

Finally, the presence of comorbidities—including opioid use, malnutrition, and low socioeconomic status—likely exacerbated infection severity and complicated management. These factors are increasingly recognized as contributors to poor outcomes in postpartum sepsis, highlighting the importance of comprehensive patient assessment and multidisciplinary care in high‐risk populations [8, 13].

## 4. Conclusion

This case adds to the evolving literature by describing an uncommon presentation of postpartum septic shock after vaginal delivery that was unresponsive to aggressive medical treatment and required operative source control. It underscores the importance of early multidisciplinary assessment, identification of high‐risk features, and decisive surgical management when conservative measures fail.

## Funding

No funding was received for this manuscript.

## Ethics Statement

This study was approved by the Research Committee of Isfahan University of Medical Sciences. Written informed consent was obtained from the patient for the publication of this case report and any accompanying images.

## Conflicts of Interest

The author declares no conflicts of interest.

## Data Availability

The author confirms that all data generated during this study are included in this published article.

## References

[1] World Health Organization , Fact Sheet of Maternal Mortality, 2016, World Health Organization, [Cited 10 Mar 2019].

[2] Bakhtawar S. , Sheikh S. , Qureshi R. , Hoodbhoy Z. , Payne B. , Azam I. , von Dadelszen P. , and Magee L. , Risk Factors for Postpartum Sepsis: A Nested Case-Control Study, BMC Pregnancy and Childbirth. (2020) 20, no. 1, 10.1186/s12884-020-02991-z, 32410594. PMC7227107 32410594

[3] Vaught A. J. , Maternal Sepsis, Seminars in Perinatology. (2018) 42, no. 1, 9–12, 10.1053/j.semperi.2017.11.003, 29463391. 29463391 PMC8015781

[4] Acosta C. D. , Knight M. , Lee H. C. , Kurinczuk J. J. , Gould J. B. , and Lyndon A. , The Continuum of Maternal Sepsis Severity: Incidence and Risk Factors in a Population-Based Cohort Study, PLoS One. (2013) 8, no. 7, e67175, 10.1371/journal.pone.0067175, 23843991. 23843991 PMC3699572

[5] Bauer M. E. and Pacheco L. D. , Sepsis and Septic Shock During Pregnancy and Postpartum, Obstetrics & Gynecology. (2025) 146, no. 2, 207–222, 10.1097/AOG.0000000000005991, 40570351. 40570351 PMC12270757

[6] Dohare R. , Roy S. , Arya A. , and Parmar S. , Sepsis in Obstetrics Score: Predicting Morbidity Among Antenatal and Postnatal Women, Bioinformation. (2025) 21, no. 7, 2090–2095, 10.6026/973206300212090, 41170080. 41170080 PMC12569875

[7] Shields A. D. , Plante L. A. , Pacheco L. D. , and Louis J. M. , Society for Maternal-Fetal Medicine Consult Series #67: Maternal Sepsis, American Journal of Obstetrics and Gynecology. (2023) 229, no. 3, B2–B19, 10.1016/j.ajog.2023.05.019. 37236495

[8] Watt S. , Lanotte P. , Mereghetti L. , Moulin-Schouleur M. , Picard B. , and Quentin R. , Escherichia coli Strains From Pregnant Women and Neonates: Intraspecies Genetic Distribution and Prevalence of Virulence Factors, Journal of Clinical Microbiology. (2003) 41, no. 5, 1929–1935, 10.1128/JCM.41.5.1929-1935.2003, 12734229. 12734229 PMC154741

[9] Surgers L. , Valin N. , Carbonne B. , Bingen E. , Lalande V. , Pacanowski J. , Meyohas M. C. , Girard P. M. , and Meynard J. L. , Evolving Microbiological Epidemiology and High Fetal Mortality in 135 Cases of Bacteremia During Pregnancy and Postpartum, European Journal of Clinical Microbiology & Infectious Diseases. (2013) 32, no. 1, 107–113, 10.1007/s10096-012-1724-5, 22907333. 22907333

[10] Moffett K. S. , Fuller K. A. , Hailemichael E. , Sico R. , Snedegar R. L. , Cummings K. F. , and Varvoutis M. S. , Lemierre′s Syndrome of the Uterus: A Case of Fusobacterium necrophorum and Streptococcus anginosus Septic Postpartum Endomyometritis, Pediatric Infectious Disease Journal. (2026) 45, no. 1, e19–e21, 10.1097/INF.0000000000004979, 40966739. 40966739

[11] Liang A. , Idowu M. B. , Eskind S. J. , and Patel S. S. , Necrotizing Fasciitis Post-Cesarean Section Leading to Transabdominal Hysterectomy, American Journal of Perinatology Reports. (2024) 14, no. 3, e235–e238, 10.1055/a-2414-7696, 39351244. 39351244 PMC11442013

[12] Alamoudi R. , Alenazi H. , Mohammed Yaqoob Ahmad H. , and Bahkali N. , Uterine Dehiscence and Abdominal Wall Necrotizing Fasciitis Following Cesarean Section: A Case Report, American Journal of Case Reports. (2025) 26, no. 26, e948791, 10.12659/AJCR.948791. 41069092 PMC12519892

[13] Habetamu T. , Abdeta T. , Debella A. , Eyeberu A. , and Yadeta T. A. , Determinants of Puerperal Sepsis Among Postpartum Women Admitted to Harar Town Public Hospitals in Eastern Ethiopia: An Unmatched Case-Control Study, BMC Womens Health. (2025) 25, no. 1, 10.1186/s12905-025-03649-8, 40089735. PMC11909991 40089735

